# Simulation Analysis and Experimental Study of Piezoelectric Power Generation Device Based on Shape Memory Alloy Drive

**DOI:** 10.1155/2022/1236270

**Published:** 2022-01-10

**Authors:** Xiaochao Tian, Zhicong Wang, Sida Zhang, Shenfang Li, Jinlong Liu, Jiaying Lin, Feifei Wang, Zhigang Yang, Jinzhi Zhu

**Affiliations:** ^1^School of Mechanical and Vehicle Engineering, Changchun University, Changchun, Jilin, China 130022; ^2^School of Mechanical and Aerospace Engineering, Jilin University, Changchun, Jilin, China 130025

## Abstract

In order to solve the problem of waste heat collection from energy consumption, a thermal energy generation device combining shape memory alloy and piezoelectric materials has been designed. The shape memory alloy is heated and deformed to drive the drive wheel continuously, and the impact wheel is deformed against the piezoelectric cantilever beam during the rotation of the drive wheel to generate electricity. In this paper, the impact force generated by the impact wheel and the output voltage of the piezoelectric cantilever beam during the rotation process are given. Finally, the experimental test shows that the larger the radius of the drive wheel, the lower the impact force of the wheel and the lower the output voltage of the piezoelectric cantilever beam; the larger the diameter of the shape memory alloy wire, the higher the impact force of the wheel and the higher the output voltage of the piezoelectric cantilever beam; the more teeth of the drive wheel, the higher the impact frequency of the piezoelectric cantilever beam and the higher the output voltage. The maximum output voltage of the thermoelectric converter is 14.2 V, when the drive wheel radius is 13 mm, the shape memory alloy wire diameter is 1 mm and the number of impact wheel teeth is 6. The new structural design provides a new structural model for waste heat recovery and thermal energy generation technology. The new structural design provides a new approach and idea for waste heat recovery and thermal energy generation technology.

## 1. Introduction

The waste heat generated by energy consumption in people's daily production and life is discharged in the form of waste heat and hot water, etc. The emission form is complicated and cannot be effectively utilized on a large scale, which not only wastes a lot of energy but also pollutes the environment, but also does not conform to the current social development requirements of low carbon and environmental protection, energy saving, and consumption reduction, and needs to be changed urgently. At present, people's research on intelligent materials has been more in-depth, with piezoelectric [1–4], electrostatic [5, 6], electromagnetic [7–9], shape memory alloy [10, 11], and other intelligent materials based on the absorption of waste heat, and power generation research is widely carried out, forming a variety of secondary energy reuse technology and methods, showing a great situation of vigorous development.

At present, the main thermal power generation technologies are temperature difference power generation [12–14], pyroelectric power generation [15–17], thermomagnetic power generation [18, 19], and thermoelastic power generation [11, 20], but they all will be restricted by some conditions and not suitable for wide application in waste heat power generation systems. The former can convert thermal energy into stress-strain and has a strong thermal-mechanical energy conversion capability, while the latter can convert stress-strain into electrical energy and has been applied to wireless networks, MEMS devices, and self-powering of portable equipment. Both materials are easy to obtain, low cost, and pollution-free. If the two are combined, they can be applied to waste heat recovery power generation by utilizing the advantages of both for thermal energy generation.

This paper combines the advantages of shape memory alloy wires and piezoelectric materials to design a new thermoelectric conversion structure based on existing materials. The structure is designed to convert thermal energy into mechanical energy using shape memory alloys and then convert mechanical energy into electrical energy using piezoelectric materials. The structural parameters of the thermoelectric conversion device are investigated in terms of their influence on the output voltage of the piezoelectric oscillator, providing a new idea and a new method for small-scale waste heat recovery and power generation technology.

## 2. Structural Design and Generator Theory

### 2.1. Structural Design

**Figure 1:**
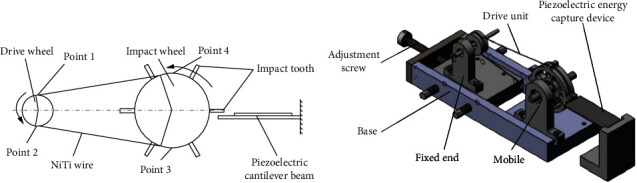
Schematic diagram of thermoelectric conversion device.

The principle of the device for thermoelectric conversion is shown in Figure 1, which mainly consists of shape memory alloy (Ni-Ti alloy), piezoelectric cantilever beam, driving wheel, and impact wheel. When the temperature rises above the As point of austenite phase change, the shape memory alloy wire undergoes a reverse martensite phase change inside the wire, the length of the shape memory alloy wire shrinks and deforms from bending to straightening, the shape memory alloy wire generates torque to the drive wheel, causing the drive wheel to rotate, the drive wheel rotation will drive the shape memory alloy wire displacement, and the displacement of the shape memory alloy wire will drive the impact wheel connected to it to rotate. After the shape memory wire leaves the heating area, the length of the Ni-Ti wire, which is in the martensite state, will recover, while the martensite in the heating area will continue to transform into austenite, and the Ni-Ti wire will shrink, making the drive wheel and the impact wheel rotate continuously. The rotation of the impact wheel will impact the piezoelectric cantilever beam, causing the piezoelectric cantilever beam to deform and generate electrical energy, in the process completing the conversion of thermal energy to electrical energy.

### 2.2. Generator Theory Analysis

**Figure 2:**
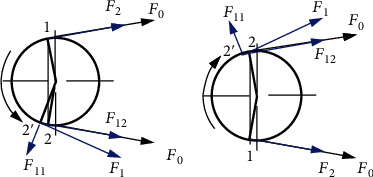
Force analysis diagram of drive components.

The force analysis diagram of the driving parts is shown in Figure 2. The shape memory alloy wire is made to contact with the drive wheel and the impact wheel by a certain preload, and the shape memory alloy wire has an initial preload force *F*_0_. The shape memory alloy wire is bent in the range of the wrap angle on the two wheels. When the heating starts at the lower end of the drive wheel and the phase change temperature is reached at point 2, at this time, the shape memory alloy wire starts to straighten due to the shape memory effect, causing the contact point between the shape memory alloy wire and the drive wheel side to move from point 2 to 2′. As the shape memory alloy wire shrinks when heated, the lower side of the shape memory alloy wire is the tight edge, while the force *F*_1_ of the tight edge decomposes *F*_12_ and *F*_11_, *F*_12_ is in the same direction as *F*_0_, and *F*_11_ is perpendicular to the direction of *F*_0_. As the shape memory alloy starts to straighten from bending resulting in a downward shift of its contact point, the force *F*_11_ generates a torque *T*_*n*_, which causes the drive wheel to drive the whole device to rotate. At the same time, the initial preload force of the shape memory alloy wire increases from *F*_0_ to *F*_12_, and the preload force of the loose side narrows from *F*_0_ to *F*_2_. At this time, the effective tension force of the shape memory alloy wire is *F*_12_ − *F*_2_, which makes the shape memory alloy wire rotate together with the two wheels under the action of the effective tension force as shown in Figure 2(a). Similarly, it can be seen that when the heating position changes, the position of reaching the phase change temperature point also changes, as shown in Figure 2(b), and its rotation direction also changes.

### 2.3. Kinetic Analysis

The kinetic analysis of the impact wheel during rotation is carried out from the perspective of energy absorption and release of shape memory alloy wire. Analysis of the boundary points between the shape memory alloy and the contact points of the two wheels is carried out.

The strain energy per unit time of the shape memory alloy wire at drive wheel point 2 is

(1) $$\begin{array}{c} W=\frac{E_{A}\pi d^{4}}{64r^{2}}\cdot r\omega_{1}. \end{array}$$

Then, the strain energy of the shape memory alloy wire at drive wheel point 2 is

(2) $$\begin{array}{c} W=\int_{0}^{d/2}\frac{1}{2}E_{A}{\left(\frac{x}{r}\right)}^{2}\cdot 2\pi xdx\cdot r\omega_{1}, \end{array}$$

where equation (1/2)*E*_*A*_(*x*/*r*)^2^ is the distortion energy per unit volume of shape memory alloy wire, 2*πxdx* · *rω*_1_ is the volume of shape memory alloy wire moving per unit time, *E*_*A*_ is the modulus of elasticity of shape memory alloy wire when heated, *r* is the radius of driving wheel, *d* is the diameter of shape memory alloy wire, and *ω*_1_ is the angular speed of the drive wheel.

In time Δ*t*, the strain energy obtained by the shape memory alloy wire is

(3) $$\begin{array}{c} W=\frac{\pi d^{4}}{64}\left(\frac{E_{A}-E_{M}}{r^{2}}-\frac{2E_{M}}{R^{2}}\right)R\omega . \end{array}$$

In time Δ*t*, the kinetic energy of the two wheels changes as follows:

(4) $$\begin{array}{c} E_{k}=\frac{1}{2}J_{1}{\left(\omega_{1}+\Delta \omega_{1}\right)}^{2}+\frac{1}{2}J_{2}{\left(\omega +\Delta \omega \right)}^{2}-\left(\frac{1}{2}J_{1}{\omega_{1}}^{2}+\frac{1}{2}J_{2}\omega^{2}\right). \end{array}$$

Since the two wheels are connected by a shape memory alloy wire, we have *rω*_1_ = *Rω*, *r* and *ω*_1_ are the radius and angular velocity of the driving wheel, and *R* and *ω* are the radius and angular velocity of the driven wheel and substitute them into (4) to obtain

(5) $$\begin{array}{c} E_{k}=\frac{1}{2}J_{1}{\left(\frac{R}{r}\right)}^{2}\left(2\omega \cdot \Delta \omega +\Delta \omega^{2}\right)+\frac{1}{2}J_{2}\left(2\omega \cdot \Delta \omega +\Delta \omega^{2}\right), \end{array}$$

where *J*_1_ is the rotational inertia of the drive wheel and *J*_2_ is the rotational inertia of the impact wheel.

According to the law of conservation of energy, the deformation energy of the shape memory alloy wire is converted into the kinetic energy of the driving wheel and the impact wheel and the friction loss between the two wheels and the bearing seat, as well as the friction between the bearing and the shaft supporting the bearing and the heat loss. Neglecting the friction loss and heat dissipation, we can get

(6) πd4R64EA−EMr2−2EMR2ω·Δt=JRr12+J2ω·Δω+12J1Rr2Δω2+12J2Δω2.

Both sides of the equation are divided by *ω* · Δ*t*

(7) πd4R64EA−EMr2−2EMR2=JRr12+J2ΔωΔt+12J1Rr2ΔωΔtΔωω+12J2ΔωΔtΔωω.

Let Δ*t*⟶0, Δ*ω*⟶0, we know that

(8) $$\begin{array}{c} \frac{d\omega }{dt}=\frac{\left(\pi d^{4}R/64\right)\left(\left(\left(E_{A}-E_{M}\right)/r^{2}\right)-\left(2E_{M}/R^{2}\right)\right)}{\left\{J_{1}{\left(R/r\right)}^{2}+J_{2}\right\}}. \end{array}$$

In order to calculate the magnitude of the impact force generated by the impact teeth during rotation, as shown in Figure 1(a), assume that the shape memory alloy line moves from point 2 to point 3 and the impact wheel makes a uniform variable speed curve motion, according to the mechanical calculation formula:

(9) $$\begin{array}{c} \omega =\frac{d\theta }{dt},\\ \xi =\frac{d^{2}\theta }{{dt}^{2}}=\frac{d\omega }{dt}. \end{array}$$

The tangential angular acceleration can be introduced

(10) $$\begin{array}{c} \xi =\frac{\left(\pi d^{4}R/64\right)\left(\left(\left(E_{A}-E_{M}\right)/r^{2}\right)-\left(2E_{M}/R^{2}\right)\right)}{\left\{J_{1}{\left(R/r\right)}^{2}+J_{2}\right\}}. \end{array}$$

Tangential acceleration

(11) $$\begin{array}{c} a_{\tau }=\left(R+X\right)\overset{••}{\theta }. \end{array}$$

Impact teeth generated during the rotation of the impact wheel

(12) $$\begin{array}{c} F_{\tau }=Ma_{\tau }=MR\frac{\left(\pi d^{4}R/64\right)\left(\left(\left(E_{A}-E_{M}\right)/r^{2}\right)-\left(2E_{M}/R^{2}\right)\right)}{\left\{J_{1}{\left(R/r\right)}^{2}+J_{2}\right\}}. \end{array}$$

The voltage is generated by the double wafer cantilever beam piezoelectric oscillator under the impact force *F* of the impact wheel [21].

(13) $$\begin{array}{c} V\left(F\right)=-\frac{3\alpha \left(1-\alpha \right)\beta g_{31}L}{Awh}F, \end{array}$$

in the formula

(14) $$\begin{array}{c} \alpha =\frac{h_{m}}{h},\\ \beta =\frac{E_{m}}{E_{p}},\\ A=\alpha^{4}{\left(1-\beta \right)}^{2}-2\alpha \left(2\alpha^{2}-3\alpha +2\right)\left(1-\beta \right)+1. \end{array}$$

*h*_*m*_ is the thickness of the metal substrate, *h* is the height of the piezoelectric beam, *E*_*m*_ is the modulus of elasticity of the metal substrate, *E*_*p*_ is the modulus of elasticity of the piezoelectric ceramic, *g*_31_ is the piezoelectric voltage constant of the piezoelectric material, *L* is the length of the piezoelectric cantilever beam, and *w* is the width of the cantilever beam.

From the above equation, the impact force of the impact wheel is related to the diameter of the shape memory alloy wire and the radius of the driving wheel. When the material is certain, the output voltage of the piezoelectric cantilever beam is related to the applied load and size.

## 3. Simulation Analysis

**Figure 3:**
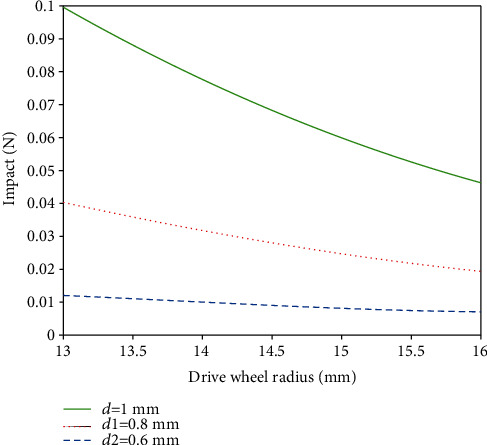
Relationship between impact force and drive wheel radius and shape memory alloy wire diameter.

The impact force was derived for different memory alloy wire diameters at different drive wheel radii by plotting the MATLAB simulation for drive wheel radii of 13-17 mm, impact tooth length of 5 mm, and thickness of 3 mm, and the curve relationship was derived as shown in Figure 3.

As can be seen from Figure 3, when the radius of the drive wheel is 13 mm, the maximum impact force generated by the impact teeth reaches 0.1 N. As the radius of the drive wheel increases, the impact force generated decreases, and the larger the diameter of the shape memory alloy wire, the greater the force generated.

**Figure 4:**
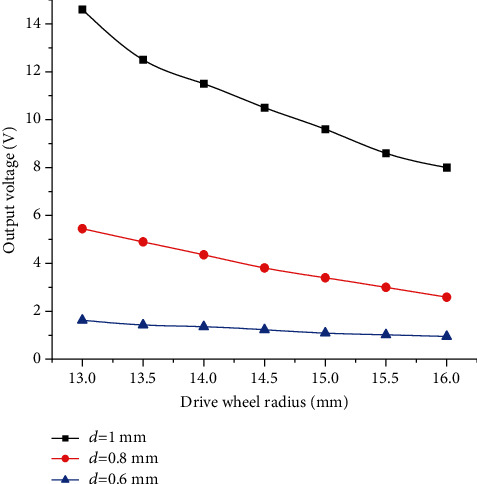
Output voltage versus drive wheel radius and shape memory alloy wire diameter.

The data of the impact force of the impact wheel corresponding to different diameters of shape memory alloy wire and the radius of the driving wheel are obtained by using MATLAB simulation, and the simulation analysis simulates the corresponding output voltage magnitude of the piezoelectric cantilever beam at the output end. The specific parameters of the selected dual-chip piezoelectric cantilever beam are shown in Table 1, and the curves are plotted as shown in Figure 4.

## 4. Experimental Study

### 4.1. Experimental Setup

**Figure 5:**
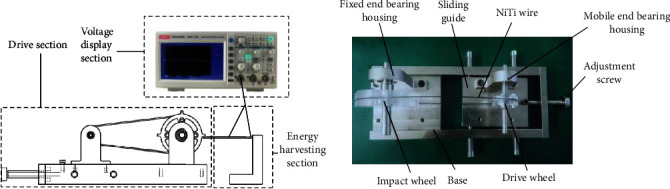
Experimental test setup diagram.

The thermoelectric power generation experiment is mainly conducted by heating the ring-shaped shape memory alloy wire in contact with the driving wheel in the driving device, which makes the impact wheel continuously rotate and hit the piezoelectric cantilever beam. The piezoelectric cantilever beam is connected to the wire, and the two ends of the wire are connected to the probe of the oscilloscope, which displays the open-circuit voltage generated by the piezoelectric cantilever beam under the impact of the impacting teeth. The schematic diagram and physical diagram of the experimental test setup are shown in Figure 5.

The thermoelectric conversion experiment is to study the impact force of the impact wheel teeth in relation to the radius of the driving wheel and the diameter of the shape memory alloy wire. The mechanical energy of the impact force of the driving part to the piezoelectric cantilever beam can be converted into electrical energy by the positive piezoelectric effect, and the amplitude of the impact force of the driving part can be understood by testing the electrical energy output of the piezoelectric cantilever beam.

### 4.2. Relationship between Driving Wheel Radius and Output Voltage of Piezoelectric Cantilever Beam

**Figure 6:**
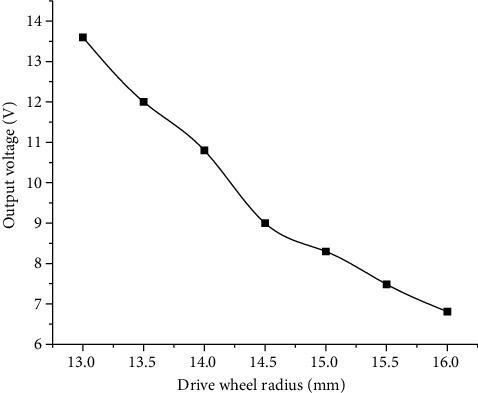
Effect of drive wheel radius on output voltage of piezoelectric cantilever beam.

The phase change temperature of the shape memory alloy is 60°C. The shape memory alloy is heated with a heat gun at 80°C. Different driving wheels with a shape memory alloy wire diameter of 1 mm and radii of 13 mm, 14 mm, 15 mm, and 16 mm are taken to test the output voltage of the piezoelectric cantilever beam at the output end (In this experiment, the center distance between the driving wheel and the impact wheel is fixed at 150 mm, the diameter of the impact wheel is 60 mm, the number of teeth is 4, the thickness of the teeth is 3 mm, and the room temperature is 20°C.). The experimental test results are shown in Figure 6.

It can be seen from Figure 6 that the range of the center distance of the two wheels rotating is different when the radius of the drive wheel is different. When the driving wheel radius is 13 mm, its center distance can rotate when it is larger than the theoretical value, while when the driving wheel radius increases, the center distance that both wheels can rotate is smaller than the theoretical value, so the larger the driving wheel radius is, the smaller the output voltage of the piezoelectric cantilever beam is.

### 4.3. Relationship between Shape Memory Alloy Wire Diameter and Output Voltage of Piezoelectric Cantilever Beam

**Figure 7:**
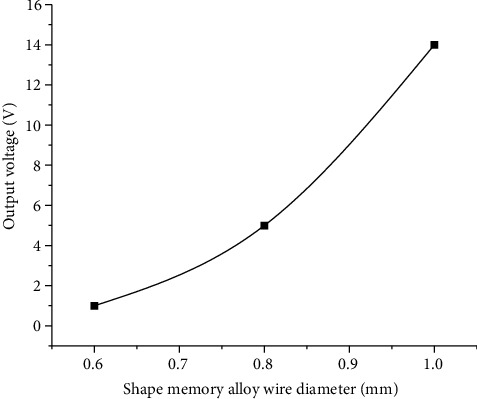
Effect of shape memory alloy wire diameter on output voltage of piezoelectric cantilever beam.

The shape memory alloy wires of different diameters of 1 mm, 0.8 mm, and 0.6 mm were heated by a heat gun at 80°C to test the output voltage of the piezoelectric cantilever beam at the output end (In this experiment, the center distance between the driving wheel and the impact wheel is fixed at 150 mm, the radius of the driving wheel is 13 mm, the diameter of the impact wheel is 60 mm, the number of teeth is 4, the tooth thickness is 3 mm, and the room temperature is 20°C.). The experimental test results are shown in Figure 7.

It can be seen from Figure 7 that the conversion efficiency of the thermoelectric conversion device is different for different diameters of the shape memory alloy wire. When the diameter of the shape memory alloy wire is 1 mm, the conversion rate of the thermoelectric conversion device is significantly higher than that of the other two diameters of the shape memory alloy wire, and the larger the diameter in a certain range, the higher the output voltage of the piezoelectric cantilever beam.

### 4.4. Relationship between the Number of Teeth of the Impact Wheel and the Output Voltage of the Piezoelectric Cantilever Beam

**Figure 8:**
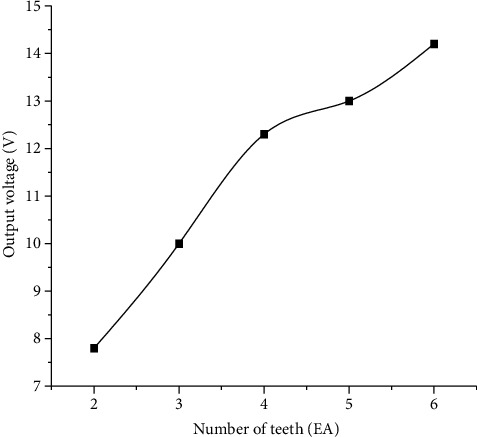
Effect of the number of teeth of the impact wheel on the output voltage of the piezoelectric cantilever beam.

The number of impact teeth mainly affects the vibration frequency of the piezoelectric cantilever beam when the impact teeth are used to continuously impact the piezoelectric cantilever beam. In the experiment, the diameter of shape memory alloy wire is 1 mm, the radius of driving wheel is 13 mm, the diameter of impact wheel is 60 mm, the length of impact teeth is 5 mm, and the thickness of impact teeth is 3 mm, and the experimental test results are shown in Figure 8.

From Figure 8, it can be seen that when the number of teeth of the impact wheel is larger, the higher the frequency of the impact wheel hitting the piezoelectric cantilever beam, the higher the output voltage of the piezoelectric cantilever beam; in the number of teeth which is 6, the output voltage of the piezoelectric cantilever beam reaches 14.2 V. Due to the manufacturing process and the limited diameter of the impact wheel, the number of teeth of the impact wheel in this paper will not be increased.

## 5. Conclusion

In this paper, we designed a device that combines shape memory alloy and piezoelectric material to form thermal power generation and conducted theoretical analysis and experimental tests on it. The following conclusions are drawn:

1. The data derived from the theoretical model by simulation match the specific experimental data
2. In the case of the same center distance between the driving wheel and the impact wheel, the larger the radius of the driving wheel, the smaller the output voltage of the thermoelectric conversion device; different diameters of the shape memory alloy wire thermoelectric conversion rate are also different; in a certain diameter range, the larger the diameter of the piezoelectric cantilever beam output voltage is higher; other factors are the same, the more teeth of the impact wheel of the driving device, the higher the frequency of impact piezoelectric cantilever beam, the higher the output voltage of the thermoelectric conversion device. The higher the output voltage of the device
3. The combination of shape memory alloy and piezoelectric material for thermoelectric conversion device is not only theoretically feasible but also has a very promising application. At the same time, it provides a new idea and a new method for thermoelectric power generation technology

## Figures and Tables

**Table 1:** Parameters of a double-chip piezoelectric cantilever beam.

Materials	Piezoelectric constants *g*_31_ × 10^‐3^	Young's modulus *E*_*p*_ (Gpa)	Poisson's ratio	Vacuum dielectric constant *ε*_0_ × 10^‐12^	Dimension (*L*∗*W*∗Th)
PZT-5H	9.11	60.6	0.34	8.85	40 mm∗20 mm∗0.2 mm
Cu	/	115	0.31	/	60 mm∗20 mm∗0.2 mm

## Data Availability

Data are available on request to the authors.
